# Loss of WSTF results in spontaneous fluctuations of heterochromatin formation and resolution, combined with substantial changes to gene expression

**DOI:** 10.1186/1471-2164-14-740

**Published:** 2013-10-29

**Authors:** Ashley E Culver-Cochran, Brian P Chadwick

**Affiliations:** 1 Department of Biological Science, Florida State University, Tallahassee, FL, USA

## Abstract

**Background:**

Williams syndrome transcription factor (WSTF) is a multifaceted protein that is involved in several nuclear processes, including replication, transcription, and the DNA damage response. WSTF participates in a chromatin-remodeling complex with the ISWI ATPase, SNF2H, and is thought to contribute to the maintenance of heterochromatin, including at the human inactive X chromosome (Xi). WSTF is encoded by *BAZ1B*, and is one of twenty-eight genes that are hemizygously deleted in the genetic disorder Williams-Beuren syndrome (WBS).

**Results:**

To explore the function of WSTF, we performed zinc finger nuclease-assisted targeting of the *BAZ1B* gene and isolated several independent knockout clones in human cells. Our results show that, while heterochromatin at the Xi is unaltered, new inappropriate areas of heterochromatin spontaneously form and resolve throughout the nucleus, appearing as large DAPI-dense staining blocks, defined by histone H3 lysine-9 trimethylation and association of the proteins heterochromatin protein 1 and structural maintenance of chromosomes flexible hinge domain containing 1. In three independent mutants, the expression of a large number of genes were impacted, both up and down, by WSTF loss.

**Conclusions:**

Given the inappropriate appearance of regions of heterochromatin in *BAZ1B* knockout cells, it is evident that WSTF performs a critical role in maintaining chromatin and transcriptional states, a property that is likely compromised by WSTF haploinsufficiency in WBS patients.

## Background

X chromosome inactivation (XCI) is the process whereby females balance the levels of X-linked gene expression with males [1], and is an archetypal example of epigenetic regulation. Much is known about the early stages of XCI, including counting, choice, initiation, and spreading of the inactivation signal [2], but our understanding of how chromatin of the chosen inactive X chromosome (Xi) and gene silencing is faithfully maintained throughout subsequent somatic cell divisions is less well understood.

The human Xi is primarily composed of two spatially distinct types of heterochromatin [3]; one is characterized by histone H3 trimethylated at lysine 9 (H3K9me3) [4,5] and association of heterochromatin protein 1 (HP1) [6], whereas the other is defined by histone H3 trimethylated at lysine 27 (H3K27me3) [7,8], elevated levels of the histone variant macroH2A [9-11], and association of the X-inactive specific transcript (XIST) [12-14]. We have previously shown that the WSTF-ISWI chromatin remodeling complex (WICH) transiently associates with the human Xi as the chromosome is undergoing DNA replication [15] and, therefore, is a candidate for maintaining this chromatin organization. Current models suggest that Williams syndrome transcription factor (WSTF), one of two subunits in the WICH chromatin remodeling complex [16], assists in reforming chromatin states of the parental cell post DNA replication [17]. Consistent with this model, depletion of WSTF protein levels by RNA-interference (RNAi) resulted in aberrant heterochromatin formation [18].

WSTF is encoded by the gene *BAZ1B*, which is located at 7q11.23 [GenBank: NG027679] [19]. It is a multifaceted protein with ties to several cellular processes, including transcription [20], replication [16], and the DNA damage response [21]. In addition to the WICH complex, WSTF has been identified as a participant in the B-WICH complex, which is associated with transcription of the ribosomal genes [22]. WSTF is also linked to the DNA damage response pathway, and was recently shown to possess tyrosine kinase activity, phosphorylating Tyr142 of H2A.X [21]. The significance of this covalent histone modification sits at the juncture between deciding on DNA repair or apoptosis [23] based on whether the modification is removed to allow access to the MDC1 complex or retained resulting in activation of the JNK pathway [24].

Interestingly, WSTF is deficient in Williams-Beuren syndrome (WBS; OMIM#194050), an autosomal dominant genetic disorder that affects approximately 1 in 7,500 individuals [25]. The disorder is characterized by a 1.5-1.8 megabase (Mb) deletion from one copy of chromosome 7q11.23 [26]. The deletion arises *de novo*, due to low-copy repeat elements flanking either side of the break points [27], and comprises approximately twenty-eight genes [28-31]. WBS patients experience an array of symptoms including cardiac defects, cognitive impairment, hypercalcaemia, growth deficiencies, and a distinct craniofacial phenotype [32-34]. Stemming from studies performed in mouse and cell lines, WSTF has been implicated as a key contributor to many of the phenotypes seen in WBS patients, despite the number of other genes that are also deficient [16,35,36]. It is unclear, however, which specific gene deficiencies contribute to each symptom; a problem common to all contiguous gene deletion disorders.

The aim of this study was to investigate the impact of WSTF loss on the maintenance of chromatin at the human Xi, and more broadly, to develop a better understanding of how the loss of this multifaceted protein affects general cellular processes. Towards this goal, we disrupted the *BAZ1B* gene using zinc-finger nuclease-assisted gene targeting in the cell line hTERT-RPE1 (RPE1)—a telomerase immortalized [37], diploid female cell line—and isolated several independent clones that lack the WSTF protein. Findings from this study underscore the necessity of determining the role of WSTF, particularly in regards to chromatin maintenance.

## Results

### Disruption of the *BAZ1B* gene using zinc finger nuclease technology

**Figure 1:**
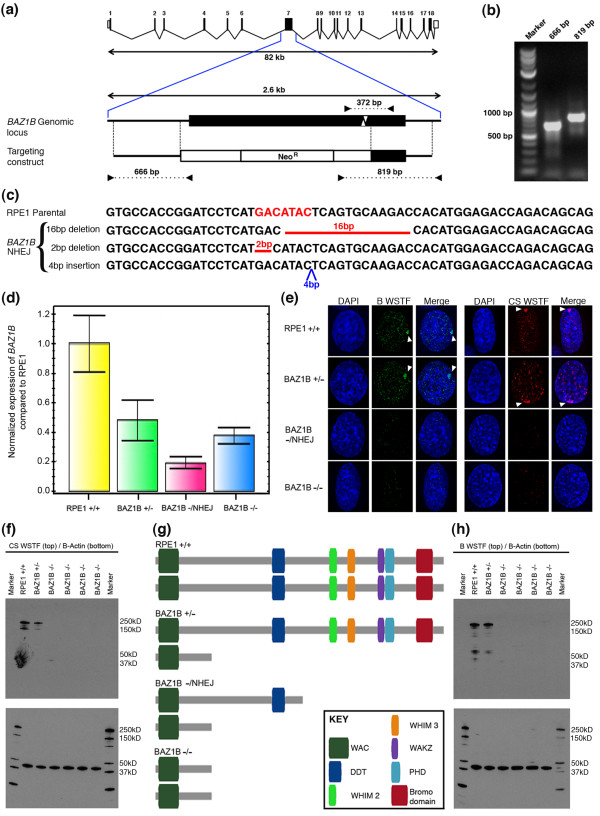
**Generation of *BAZ1B *knockout cells. (a)** Schematic representation of the *BAZ1B* gene. Exons: numbered vertical bars. Introns: connecting lines. Exon 7 is bracketed by the blue lines, and expanded beneath. The targeting construct is shown beneath, indicating the location of the neomycin resistance gene and the extent of the homology arms (regions between the vertical dotted lines). Left and right side screening PCRs are indicated below by inward-facing arrowheads. The NHEJ PCR is shown above Exon 7, and the ZFN cutting site is indicated by the white arrowheads within the exon. **(b)** Example of an ethidium bromide stained gel showing a positive clone by PCR. **(c)** DNA sequence alignment of parental RPE1 with NHEJ disrupted clones. The cutting site for the ZFNs is highlighted in red. Deletions in NHEJ clones are indicated by the red gaps; insertions by the blue triangle. **(d)** qRT-PCR analysis of *BAZ1B* showing levels in a heterozygous mutant (*BAZ1B* +/-), a clone with one allele targeted and one disrupted by NHEJ (*BAZ1B* -/NHEJ) and a clone targeted at both alleles (*BAZ1B* -/-) relative to parental RPE1. **(e)** Indirect immunofluorescence showing the distribution of WSTF in RPE1, a *BAZ1B* +/-, *BAZ1B* -/NHEJ and a *BAZ1B* -/- clone. WSTF is shown in green for the Bethyl Labs anti-WSTF antibody and red for the Cell Signaling anti-WSTF antibody. The white arrowheads indicate the location of the Xi. The nucleus is counterstained with DAPI (blue). **(f)** Western blot for WSTF with the Cell Signaling antibody in RPE1, a *BAZ1B* +/-, and various independent *BAZ1B* knockout mutants. Actin is shown immediately below. **(g)** Schematic map of WSTF showing the relative location of functional domains, and the truncated proteins possible in the different mutant types. **(h)** Western blot using the Bethyl Labs anti-WSTF antibody as described for part-**f** above.

The development of zinc finger nuclease technology provides opportunities for genomic editing at precise locations in human cells [38]. Here, we introduced into RPE1 cells a pair of zinc finger nucleases (ZFNs) designed to exon-7 of the *BAZ1B* gene, along with an engineered repair template (Figure 1a). The repair template consisted of a central promoterless neomycin open reading frame (ORF) that was preceded by a splice acceptor and internal ribosome entry site, and followed by a poly-adenylation signal [39]. This central cassette was flanked by homology arms (HA) of approximately 600–800 bp that were derived from sequences in the vicinity of exon-7 (Figure 1a). The general strategy is that upon generation of a double-stranded break by the ZFNs, the repair template is used though homology mediated repair to patch the damage, introducing the neomycin ORF in place of exon-7 in the process. This synthetic exon promoter trap (SEPT) is incorporated into the targeted *BAZ1B* transcript, prematurely truncating the message through poly-adenylation and providing neomycin resistance, driven by the gene’s own expression. Transfected cells were placed under neomycin selection, and after two weeks clones emerged.

Screening for correct targeting was achieved by performing PCR for both the left and right sides of the targeted region with a primer within the SEPT cassette and a primer located outside of the HAs at the genomic locus (Figure 1b). We screened a total of 119 single cell clones by PCR. We identified 13 clones that were correctly targeted at one or both alleles of the *BAZ1B* gene. To screen for targeting at both alleles, a second PCR was performed with primers flanking the ZFN target site (Figure 1a). If a clone was targeted at both alleles, this sequence would no longer be present and no product would be detected. Three clones were determined to be successfully targeted at both alleles. Double-strand breaks generated by ZFNs can be repaired by either homology mediated repair or non-homologous end joining (NHEJ). NHEJ is not a precise repair pathway, frequently resulting in introduction of small insertions or deletions at the target site [40], which can disrupt the reading frame of the target gene. For those clones that were targeted at only one allele, we screened for NHEJ at the second allele by sequencing the PCR product. We identified three clones that were targeted at one allele and have the second allele disrupted by NHEJ (Figure 1c), and several clones that were targeted at one allele but wild-type at the second allele. This latter group of heterozygous mutant clones are useful models to investigate the contribution of *BAZ1B* hemizygosity in WBS in the absence of the other >20 genes that are typically lost alongside *BAZ1B* in this disease, all of which potentially contribute to the phenotype. The clones used in this study and the nature of their mutation/s is indicated in Table 1.

**Table 1:** ***BAZ1B *** **mutations in clones used in this study**

**Clone**	**BAZ1B Allele 1**	**BAZ1B Allele 2**
*BAZ1B*-A6 (+/-)	Promoter trap targeted	Wild type
*BAZ1B*-D5 (-/-)	Promoter trap targeted	Promoter trap targeted
*BAZ1B*-F3 (-/-)	Promoter trap targeted	Promoter trap targeted
*BAZ1B*-M1 (-/-)	Promoter trap targeted	16 bp deletion
*BAZ1B*-N6 (-/-)	Promoter trap targeted	2 bp deletion
*BAZ1B*-O4 (-/-)	Promoter trap targeted	4 bp insertion

Table indicates the nature of the mutations generated at the two chromosome 7 *BAZ1B* alleles for the clones used in this study. Next to each clone name, (+/-) indicates heterozygous mutant whereas (-/-) indicates homozygous mutant.

Quantitative real time PCR (qRT-PCR) using primers that spanned exons-2 and -3 indicated that a clone targeted at only one allele (Clone A6), showed *BAZ1B* mRNA at 47.6% of parental cells. Clone M1, targeted at one allele and mutated at the second by NHEJ, expressed *BAZ1B* mRNA at 18.3%, whereas clone D5, that is targeted at both alleles, showed 33.7% (Figure 1d). Expression of *BAZ1B* is expected as exons 1–6 are uninterrupted and isolation of clones required *BAZ1B* expression to trap the SEPT cassette and express neomycin resistance. The reduced levels of mRNA likely represent destabilization of the *BAZ1B* mRNA.

We then employed indirect immunofluorescence (IF) to investigate the distribution of WSTF in the *BAZ1B* mutants. Here, we used two independent antibodies raised to different regions of the WSTF protein; one to the first 50 amino acids, and the other to a peptide at the carboxy terminus. Results indicate that WSTF distribution in the *BAZ1B* heterozygous mutant was very similar to that of the parental cells, whereas WSTF staining was absent in all double-targeted clones suggesting a complete knockout of the gene (Figure 1e). To confirm loss of WSTF, Western blotting was performed on protein extracts from the mutants. Consistent with the IF analysis, protein levels were reduced in the heterozygous mutant and absent from all independent homozygous mutant clones (Figure 1f). By targeting exon-7, the ORF contained within the first 6 exons could potentially generate a truncated protein of 87.3 kDa (from NHEJ targeted alleles, amino acids 1–761) or 34.5 kDa (from double targeted alleles, amino acids 1–297) (Figure 1g). The NHEJ form would contain the WAC and DDT domains only, whereas the double targeted form of the protein would only contain the WAC domain. In order to detect such a protein, we repeated the Western analysis using the second antibody that was raised to the first 50 amino acids of WSTF. Consistent with the first Western, no truncated protein could be detected (Figure 1h).

### Loss of WSTF does not impact chromatin of the human Xi

At interphase, the Xi can be readily detected as a densely staining heterochromatin mass, or Barr body, that is typically located in a perinucleolar or peripheral location in the nucleus [41]. The Xi is painted by XIST RNA [14] and facultative heterochromatin markers are readily observed due to the chromosome wide silencing, resulting in an unusually large concentration of these features. Previously, we have shown that WSTF as part of the WICH complex, associates with the Xi territory during S-phase [15] as the chromosome is replicated [42]. In order to determine if WSTF loss impacts the organization and maintenance of Xi chromatin, we assessed a number of Xi markers for changes in their distribution.

**Figure 2:**
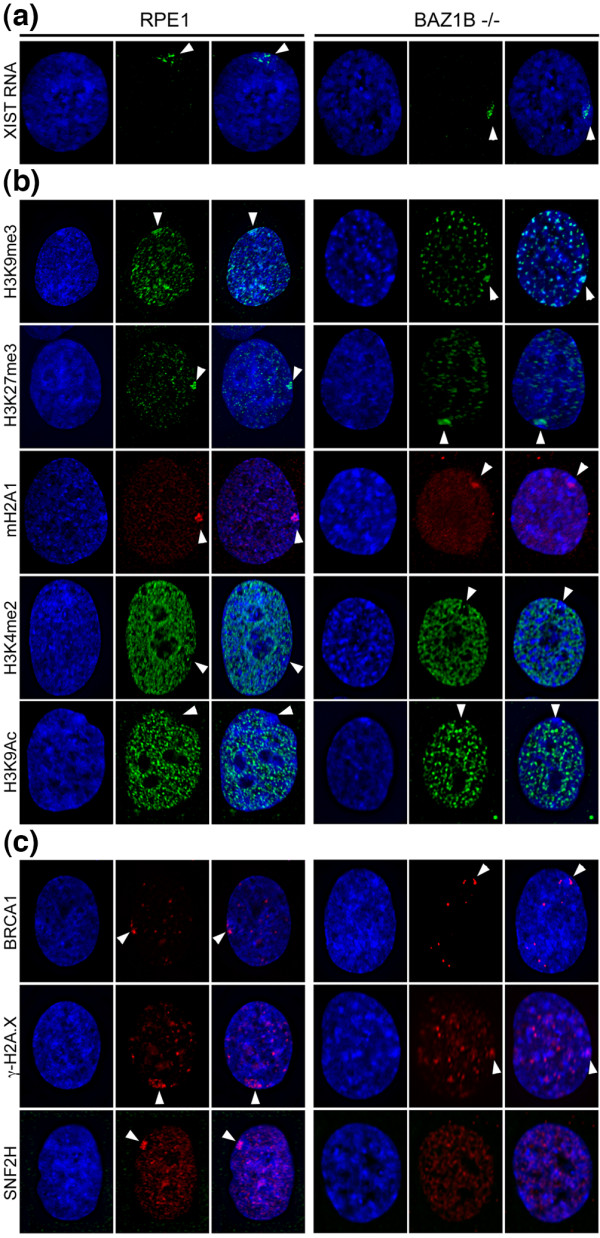
**Investigating the impact of WSTF loss on the human Xi. (a)** Distribution of XIST RNA in RPE1 and *BAZ1B* -/- using a direct-labeled RNA FISH probe (green). Nuclei in all images are counterstained with DAPI (blue), merged images are shown on the right of each group of three images, and white arrowheads indicate the location of the Xi. **(b)** Examples of the distribution of markers that are elevated at the Xi territory including H3K9me3 (green), H3K27me3 (green) and mH2A1 (red), and those that are underrepresented; H3K4me2 (green) and H3K9Ac (green). **(c)** Examples of elevated levels of BRCA1, γ-H2A.X and SNF2H at the Xi (red). SNF2H was never observed enriched at the Xi *BAZ1B* -/-.

First, we performed RNA FISH to investigate whether the Xi had retained its association with XIST RNA. Here, we found that XIST RNA was enriched in the vicinity of the Barr body in *BAZ1B* knockouts in a pattern indistinguishable from that of parental RPE1 cells (Figure 2a). Next, we examined other chromatin features of the Xi. Typical characteristics of the human Xi include an enrichment of H3K9me3 [4,5], H3K27me3 [7,8], and mH2A1 [10], and a noticeable absence of H3K4me2 [4,43] and histone acetylation [44], such as H3K9Ac. By IF, none of these features appeared perturbed in the *BAZ1B* knockouts, having distribution patterns indistinguishable from that observed in parental RPE1 cells (Figure 2b). However, of note, the distribution of H3K9me3 throughout the rest of the nucleus appeared more punctate (Figure 2b, top row).

WSTF is one of several proteins that transiently associate with the Xi during S-phase [15]. Other transient features of the Xi during this time include the association of the breast cancer gene (BRCA1) and the phosphorylation of histone H2A.X at serine 139 (γ-H2A.X) [45]. We sought to determine if these features were altered in the *BAZ1B* knockout cells. Towards this aim, we employed IF using antibodies to BRCA1, γ-H2A.X, and SNF2H—WSTF’s partner in the WICH complex [16]. We found that BRCA1 and γ-H2A.X still localize to the Xi (1.5% and 2.5% respectively, n = 200) (Figure 2c), similar to what is reported in the parental cells [15,45]. In contrast, SNF2H was no longer detected at elevated levels at the Xi (n = 1242). However, SNF2H protein still exhibited diffuse staining at other locations within the nucleus, consistent with its role in other complexes independent of WSTF and WICH [46-49].

### Cells lacking WSTF exhibit a unique nuclear phenotype

**Figure 3:**
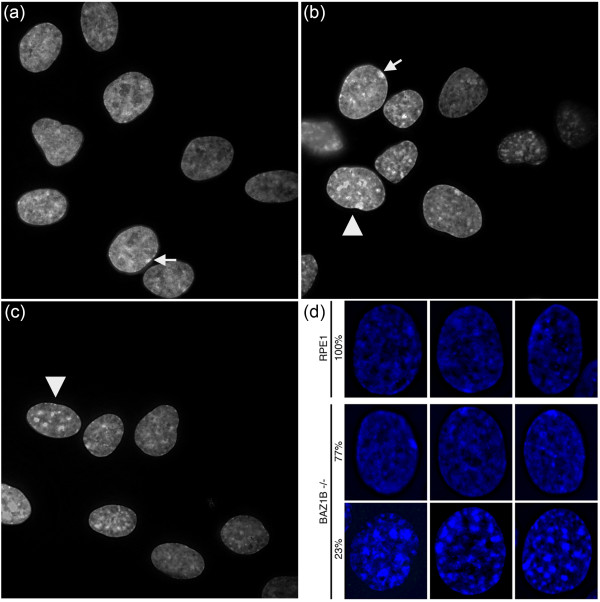
***BAZ1B *homozygous knockout cells exhibit a unique nuclear phenotype.** Representative images of RPE1 **(a)**, *BAZ1B*-N6 **(b)** and *BAZ1B*-O4 **(c)** nuclei counterstained with DAPI. White arrow-heads in **(b)** and **(c)** point to block-containing nuclei. White arrows point to the DAPI-dense Barr body. Images were captured using settings defined for RPE1 that result in pixel saturation for block-containing *BAZ1B* -/- nuclei. **(d)** RPE1 and *BAZ1B* -/- nuclei stained with DAPI showing the appearance of large DAPI-dense blocks in a fraction of mutant cells, as indicated to the left.

DNA staining with 4′,6-diamidino-2-phenylindole (DAPI) revealed a nuclear phenotype unique to *BAZ1B* knockout cells. In parental hTERT-RPE1 nuclei, the most intense DAPI staining structure corresponds to the Barr body, as indicated by the white arrow in Figure 3a. In contrast, the nuclei of *BAZ1B* knockout cells show additional DAPI dense bodies of comparable to greater intensity as demonstrated by the pixel saturation in Figure 3b and 3c. Not all nuclei show the appearance of these structures. We observed that 23.21% of nuclei (n = 2356) possessed numerous large DAPI-dense regions (Figure 3d). Intriguingly, transient reduction of either component of the WICH complex by RNAi was reported to result in a gain of heterochromatin [18], consistent with these observations.

We sought to further characterize both the composition and dynamics of the DAPI–dense regions, as the appearance of blocks in only a subset of cells raised the possibility that the blocks could be linked to a cell cycle, arrest in proliferation, or potentially early stages of cell death.

### DAPI-dense regions are enriched for constitutive heterochromatin markers

**Figure 4:**
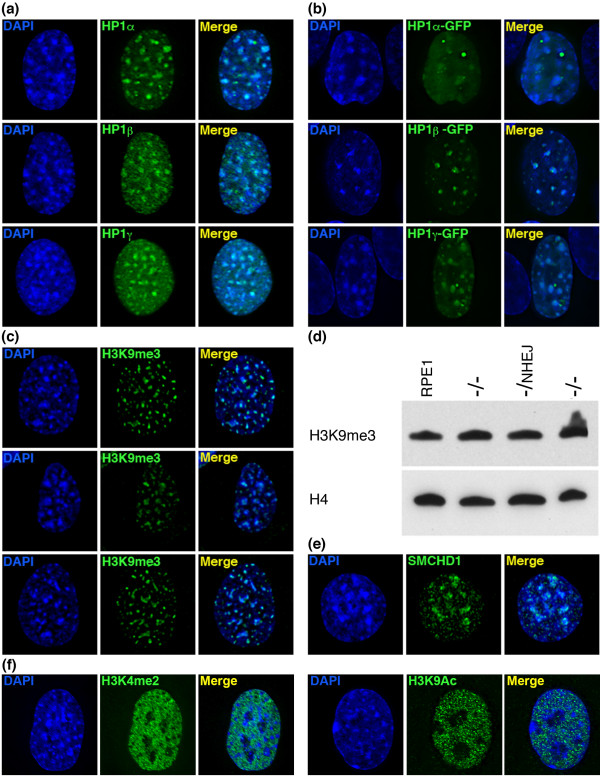
***BAZ1B *-/- DAPI-dense blocks are characterized by constitutive heterochromatin markers. (a)** Association of endogenous HP1α, HP1β, and HP1γ (green) with DAPI-dense blocks. Nuclei are counterstained with DAPI (blue) and merged images are shown in the right-side panel. **(b)** Association of exogenous GFP-tagged HP1α, HP1β, and HP1γ (green) with DAPI-dense blocks. **(c)** Examples of elevated levels of H3K9me3 (green) at DAPI-dense blocks. **(d)** Western blot showing comparable levels of H3K9me3 in mutant compared to RPE1. Anti-histone H4 is used as a loading control. **(e)** Example of the association of SMCHD1 (green) with DAPI-dense blocks. **(f)** Examples showing the lack of association of euchromatin markers H3K4me2 and H3K9Ac with the DAPI-dense blocks.

Areas of intense DAPI staining in nuclei typically correspond to regions of heterochromatin, such as the constitutive heterochromatin of mouse major satellite [50], senescence associated heterochromatin blocks [51], or facultative heterochromatin of the Xi [14]. The blocks seen in the *BAZ1B* homozygous mutants were reminiscent of the heterochromatic Barr body in both DAPI intensity and size. For this reason, we examined the blocks for association with heterochromatic features and various complexes known to interact with heterochromatin. To accomplish this, we pursued several avenues of investigation. First, we explored the relationship of the blocks with the three different HP1 proteins: HP1α, HP1β, and HP1γ [52-54]. Chromatin associated HP1 levels were shown previously to increase in cells with depleted levels of WSTF, particularly HP1β [18]. Consistent with this observation, we found that all three HP1 proteins localize to the DAPI-dense blocks (Figure 4a). To validate that all three isoforms did indeed associate with the blocks and that this was not a by-product of antibody cross-reactivity, we transiently transfected *BAZ1B* knockout cells with HP1α, HP1β, or HP1γ green fluorescent protein (GFP) expression constructs. We found that all three exogenous HP1 types, with HP1α to a lesser extent, localized to the blocks (Figure 4b).

Next, we continued to characterize the DAPI-dense blocks in the *BAZ1B* knockouts through IF to a variety of heterochromatin-associated proteins and covalent histone modifications. Our results showed that, consistent with binding of HP1 to H3K9me3 [55-57], the blocks were clearly defined by this modification (Figure 4c).

Given the size of the blocks, we wondered if this represented an overall increase in H3K9me3 levels. Histones were extracted from parental and WSTF mutant cells and H3K9me3 levels detected by Western blotting. Despite the apparent increase by IF, H3K9me3 levels were comparable between mutants and parental cells (Figure 4d). However, given that only 23% of cells show blocks, it is possible that increases are masked by the 77% of normal cells.

Recently, the structural maintenance of heterochromatin protein, SMCHD1, was linked to HP1 and H3K9me3 [58]. In agreement with this, SMCHD1 associated with the DAPI blocks (Figure 4e). With the exception of the Xi, comparable large blocks of HP1α, HP1β, HP1γ, H3K9me3 and SMCHD1 were not a feature of parental cells (Additional file 1).

Although they did not completely colocalize, areas of MBD1 and HMG-I/Y enrichment were present within portions of the blocks (Additional file 1). As expected, the blocks had no association with euchromatin markers (Figure 4f). However, 14 other heterochromatin proteins did not associate with the blocks, including TIF1B, EZH2, Mi2, RbAp46, RbAp48, SAP18, SAP30, SIN3A, SIN3B, HDAC1, HDAC2, MBD3, ATRX, and SETDB1, nor histone H1 or the histone variant macroH2A1 (Additional file 1).

### DAPI-dense blocks do not correspond to common nuclear structures

**Figure 5:**
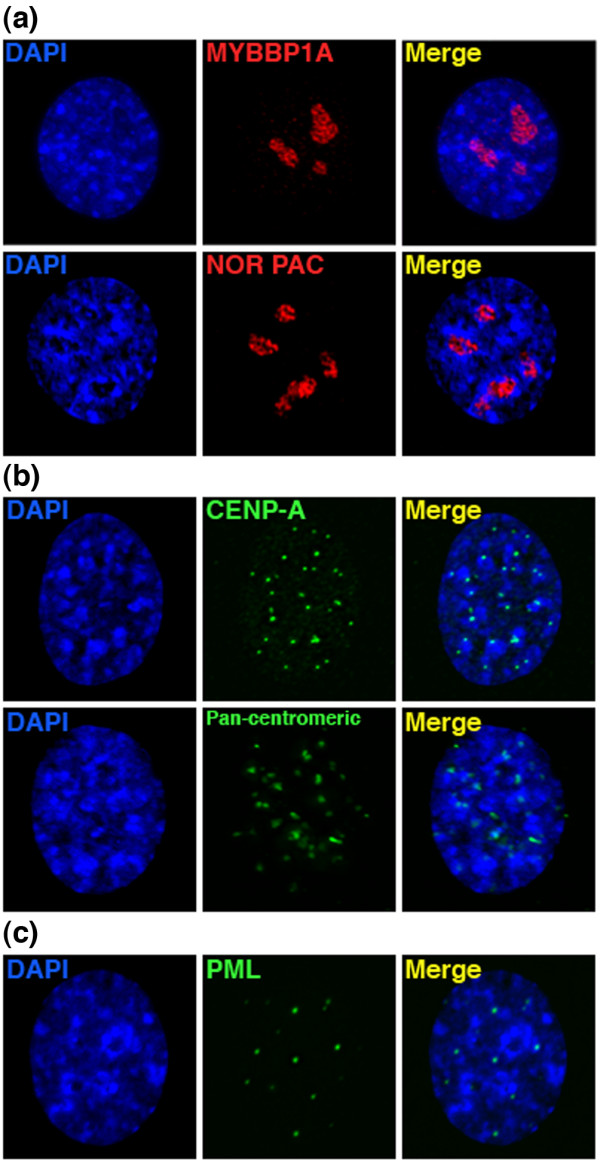
***BAZ1B *-/- DAPI-dense block relationship to other nuclear structures. (a)** DAPI-dense block distribution relative to nucleoli markers MYBBP1A (red, IF) and NOR (red, direct-labeled FISH). **(b)** DAPI-dense block distribution relative to centromere markers CENP-A (green, IF) and alpha-satellite DNA indicated by a pan-centromeric direct-labeled FISH probe (green). **(c)** DAPI-dense block distribution relative to PML bodies (green, IF).

We sought to determine if the DAPI-dense regions were associated with disruption of B-WICH activity, due to its participation in ribosomal DNA transcription at nucleoli [20]. The nucleolar organizing region (NOR) of the acrocentric chromosomes (13, 14, 15, 21, and 22) localizes to and can be used to define the extent of nucleoli [15]. We performed DNA FISH using a BAC from the NOR as a probe, and IF using an antibody raised against MYBBP1A, a member of the B-WICH complex [20], to examine if the DAPI blocks coincided with nucleoli. We found that neither NOR or MYBBP1A overlapped with the DAPI-dense blocks (Figure 5a).

Given the large number of DAPI-dense blocks, we wondered if they corresponded to the inappropriate spread of centromeric heterochromatin. The reasoning behind this assumption is that cells treated with RNAi to *BAZ1B* showed an increase in HP1β, which was postulated to represent gain of heterochromatin [18], and that HP1 is a feature of centromeric heterochromatin [59]. We addressed this two ways, first by DNA FISH with a pan-centromeric probe, and second by IF to the centromeric protein CENP-A [60,61]. We found that the blocks did not overlap with either centromeric DNA or CENP-A-positive regions (Figure 5b), although signals often resided at the edge of the blocks.

Prior studies have also linked WSTF function to several cellular processes including DNA replication (WICH), transcription (B-WICH), and epigenetic silencing (WICH) [62]. Like WSTF, promyelocytic leukemia (PML) bodies have also been linked to the processes of DNA replication, transcription, and epigenetic silencing [63]. In addition, the number, and sometimes size, of PML bodies are reminiscent of the DAPI-dense blocks that we see in *BAZ1B* knockout cells. Altogether, this led us to question whether the DAPI-dense blocks were expanded PML bodies. To test this question, we detected PML bodies by IF. We found that PML bodies had not increased in size and did not colocalize with the DAPI-dense blocks (Figure 5c).

### DAPI-dense blocks in *BAZ1B* knockout cells are not linked to and do not disrupt the cell cycle

**Figure 6:**
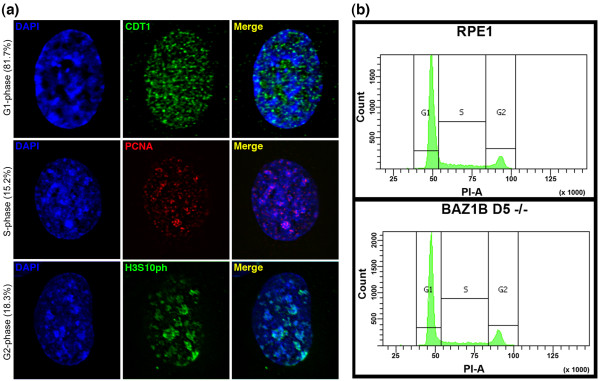
**DAPI-dense blocks in *BAZ1B *-/- are present in G1, S and G2 phase and do not disrupt the cell cycle. (a)** Examples of DAPI-dense blocks in *BAZ1B* -/- cells at different stages of the cell cycle as indicated by the appropriate markers; CDT1 for G1 (green, top), PCNA for S-phase (red, middle) and H3S10ph for G2 (green, bottom). H3S10ph accumulates first at heterochromatic regions before spreading throughout the nucleus [68], accounting for the elevated levels observed at the heterochromatin blocks in the example shown. The percentages given in brackets to the left of the DAPI image indicate the number of cells positive for the cell cycle stage marker in the population of cells examined (CTD1, n = 83; PCNA, n = 105; H3S10ph, n = 115). Note that the numbers are greater than 100%. This is because some H3S10ph positive cells are in late S-phase in RPE1 [15], and CDT1, while present throughout the cell cycle, is most abundant during G1 [64]. Consequently some cells from other stages of the cell cycle are inadvertently included in these numbers. **(b)** Representative FACS profile for RPE1 and *BAZ1B* -/-.

The appearance of the DAPI-dense blocks in only a subset of cells could be explained by their appearance being associated with a particular period during the cell cycle. In order to test this, we examined cells labeled with markers of the cell cycle: CDT1 for G1 [64,65], PCNA distribution for S-phase [66,67], and phosphorylation of histone H3 at serine 10 (H3S10ph) for G2 phase [68]. DAPI-dense blocks were detected at all stages of the cell cycle (Figure 6a).

Next, we wondered if the appearance of these blocks affected the progression of the cell cycle. However, cell cycle profiles determined by fluorescence activated cell sorting (FACS) were indistinguishable between parental RPE1 and *BAZ1B* knockout cells (Figure 6b).

These observations suggest that perhaps cells were slowly acquiring blocks over time, or that only a subset of cells always have blocks and others do not.

### Cells containing DAPI-dense blocks do not accumulate over time in culture and are not stably inherited

In order to determine if the number of cells with DAPI-dense blocks was accumulating over time, we monitored cells for the percentage of DAPI-dense positive nuclei every 10 passage doublings for a total of 70 doublings. We found that at any given passage doubling, the population of cells with DAPI-dense blocks remained consistently around 20-25% of cells (Table 2).

**Table 2:** Appearance of DAPI-dense blocks does not change with passage doubling

**Passage doubling**	**Percentage of nuclei with DAPI-dense blocks**	**Number of nuclei scored**
14	31.85	135
24	22.74	277
34	22.94	327
44	20.27	291
54	21.30	315
64	24.46	372
74	22.14	262
84	24.13	377

Table shows the percentage of BAZ1B -/- clone D5 nuclei that showed DAPI-dense blocks over the course of 70 passage doublings. A total of 2356 nuclei were scored. The mean and standard deviation is 23.73% +/-3.56.

Since only a subset of cells possessed the DAPI blocks, we questioned whether they were a stable heritable trait that could be passed on to daughter cells. If this were the case, we would expect that clones derived from a non DAPI-dense block containing cell would show no blocks, while those derived from a DAPI-dense block containing cell would all contain blocks. To investigate this question, we isolated single cell clones and examined eight subclones of each of three independent *BAZ1B* homozygous knockout mutants for the presence or absence of the DAPI-dense blocks. Interestingly, all subclones contained cells with and without DAPI-dense blocks (Table 3), which suggested that cells could develop and resolve blocks regardless of the state of the parent cell. However, it remains a possibility that those cells acquiring blocks arrest in the cell cycle and eventually die.

**Table 3:** DAPI-dense block incidence in single cell clones

**BAZ1B -/- clone**	**Percentage of nuclei with DAPI-dense blocks for 8 independent subclones**	**Mean & standard deviation**
D5	78, 50, 76, 78, 44, 58, 40, 48	59.0% +/- 16.0
F3	8, 40, 22, 56, 24, 42, 20, 24	29.5% +/- 14.0
M1	20, 28, 20, 18, 22, 20, 32, 38	24.8% +/- 7.0

Table shows the percentage of nuclei with DAPI-dense blocks for each of eight single-cell subclones derived from three independent BAZ1B -/- clones. Fifty nuclei were scored for each subclone.

### DAPI-dense blocks are dynamic

The transient transfection of HP1β into *BAZ1B* knockout cells clearly highlighted the DAPI-dense blocks (Figure 4b), providing an opportunity to monitor live cells expressing the GFP construct in real time. Cells expressing HP1β-GFP were followed over 24 hour and 72 hour periods. Since the number of block-containing cells did not increase in the population over time, the blocks could either be spontaneously forming and resolving, or those cells with blocks could arrest, or undergo apoptosis. We observed that the block-containing nuclei did not undergo apoptosis, but were readily able to divide, ruling out arrest in the cell cycle (See Additional file 2). We found that a block-containing cell was able to give rise to a daughter cell that also acquired blocks, and, therefore, the condition was mitotically heritable (Additional file 3). Furthermore, the blocks appear to resolve with the exception of the Xi-associated HP1β-GFP block, highlighting the dynamic nature of the blocks. Interestingly, we noted that, despite substantial movement by the cell body, the blocks were static within nuclei (Additional file 4), similar to observations made in real-time with replication factories [67]. Finally, blocks were observed for as long as 24 hours in cells before dividing, and appeared to resolve right before entering mitosis, confirming that they are not transient features of a particular cell cycle stage (Additional file 3). Given these observations and the fact that single cell clones give rise to a similar fraction of cells with DAPI-dense blocks as the parental cell population (and not 100% block-containing or 100% block-free), we conclude that the appearance and resolving of the blocks is a dynamic process. What triggers the formation and subsequent resolution of the blocks in some cells and not others is presently unknown.

### Introduction of exogenous full length WSTF ORF is sufficient to remove the appearance of DAPI-dense blocks

**Figure 7:**
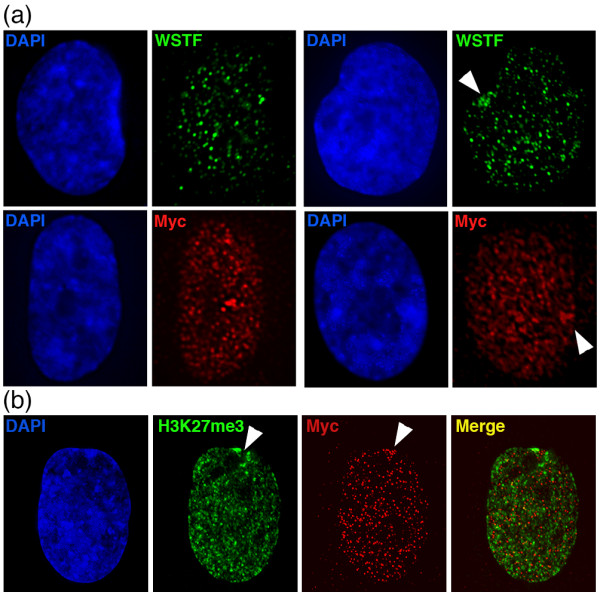
**Distribution of exogenous Myc-tagged WSTF in *BAZ1B -/- *nuclei. (a)** Distribution of exogenous WSTF as detected by anti-WSTF IF in *BAZ1B* -/- nuclei (green) (top row) and the MYC-tag (red) (bottom row). Examples of elevated levels at the Xi are indicated with the white arrowheads. **(b)**vElevated levels of exogenous WSTF-Myc (red) at the Xi as defined by H3K27me3 (green). The location of the Xi is indicated by the white arrowhead. Nuclei are counterstained with DAPI (blue).

We wondered if the DAPI-dense blocks in *BAZ1B* knockout cells could be removed by reintroducing wild type WSTF protein. To test this, an expression construct containing a Myc-tagged full-length WSTF ORF was introduced into WSTF knockout cells by Nucleofection. Cells were examined by IF using antibodies specific to the Myc-tag and WSTF. Cells that were positive for Myc were also positive for WSTF (n = 94) (Figure 7a). Furthermore, transfected cells were restored for transient association of WSTF to the Xi as defined by H3K27me3 [15] (Figure 7b). We examined Myc-positive nuclei for DAPI-dense blocks. All Myc-positive nuclei showed DAPI patterns comparable to parental RPE1, with no evidence of intense DAPI-dense blocks (n = 119), suggesting that the blocks can be resolved by reintroduction of wild-type protein.

### Loss of WSTF results in gene expression changes

**Figure 8:**
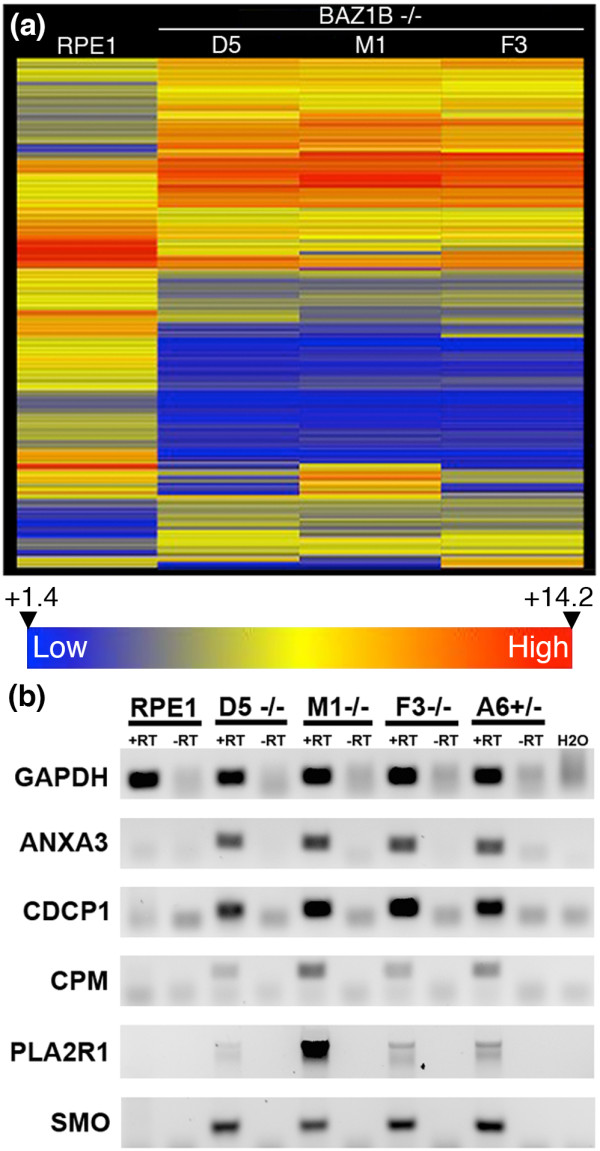
**Gene expression changes in *BAZ1B *-/- cells. (a)** Dendrogram (Heat Map) of hierarchical clustering of gene expression between RPE1 and three independent *BAZ1B* -/- clones. Heat map represents 586 data points, corresponding to probes showing greater than 2-fold change in gene expression at 95% confidence. Expression levels range from +1.4 to +14.2, corresponding to signal intensity on the microarray and represented by a blue (low) to red (high) gradation indicated by the scale bar beneath the heat map. Each column represents the averaged signal from three replicate data sets for each clone. Each row represents a single specific probe. Changes in gene expression can be seen by the difference in row colors between the mutants and parental cells. **(b)** Representative examples of genes activated in *BAZ1B* -/- cells by standard end-point RT-PCR analysis shown as an inverted image of an ethidium bromide stained agarose gel. The gene name is given to the left, and the cell line indicated at the top. Lanes show with (+RT) and without (-RT) reverse transcriptase and a water control (H2O).

Given that the DAPI-dense blocks are enriched for markers of constitutive heterochromatin, and that WSTF is involved in transcription [62], we assessed the WSTF mutant cells for changes in gene expression, with the thought that perhaps most gene expression changes would be reductions due to the gain in heterochromatin. Total RNA was extracted from three separate cultures of parental RPE1 cells as well as from three separate cultures for each of three independent *BAZ1B* knockout clones, and one heterozygous mutant clone. Residual DNA in the RNA preparation was digested and its removal confirmed by PCR before preparing cDNA, labeling and hybridizing to a NimbleGen human gene expression 12x135K array containing >45,000 transcript targets. Genes that had significant changes of two fold or more compared to RPE1 (p < 0.05) were identified for each mutant. The heat map shown in Figure 8a, clearly shows consistent and substantial changes in gene expression between the three independent *BAZ1B* mutants as compared to the parental RPE1 cells. The genes that were not shared between all three independent mutants were excluded. The remaining genes of interest can be found in Additional file 5 (n = 515). In the knockout cells, genes for which expression levels changed were not all reductions; some went up (173) while others went down (342). To validate the microarray data, we performed quantitative RT-PCR (qRT-PCR) on several genes that showed increases or decreases in the microarray data relative to the parental RPE1 cells. All consistently showed the same trend (data not shown). Interestingly, of the 515 gene expression changes observed in the *BAZ1B* knockouts, 72 of these were shared in the heterozygous mutant cells (Additional file 6). This indicates a degree of sensitivity to WSTF haploinsufficiency, further supporting an important contributing role of *BAZ1B* hemizygosity in WBS.

Despite our supposition that most genes would be silenced due to the gain in heterochromatin, as mentioned above, a substantial number of genes showed increased expression in the mutants relative to the parental cells. Among these, a subset appeared to show activation in both *BAZ1B* -/- and *BAZ1B* +/- cells (Figure 8b). These genes could not be assessed by qRT-PCR, as fold change cannot be determined when expression of a control or unknown sample is zero, but the endpoint RT-PCR analysis is consistent with the microarray data.

Gene expression changes were not restricted to certain chromosomes or chromosomal regions (Additional file 5). With the exception of chromosome 21, all chromosomes have genes that showed increases or decreases in expression, although all changes were less than 2.0% of the total gene content for the chromosome (coding and non-coding). Given that chromosome 21 has the lowest gene content, it does not seem that this chromosome is an exception for any biological reason. Despite the blocks frequently residing close to nucleoli, the NOR-containing chromosomes (13, 14, 15, 21 and 22) did not have the highest number of silenced genes. Instead, the chromosomes that contained the largest number of silenced genes were chromosomes 20 (1.55%) and 12 (1.50%). No obvious trend was observed for silenced genes concerning genomic location, such as residing close to large satellite blocks or in pericentromeric or subtelomeric regions. Interestingly, the protocadherin cluster on chromosome 5q31.3 that is associated with various neuropsychiatric disorders [69], and implicated in tumor suppressor activity [70,71], consistently showed an increase in expression of most of the alpha members, but decreases in the adjacent beta and gamma clusters. Intriguingly, in the heterozygous *BAZ1B* mutant, the alpha cluster was up-regulated, similar to the complete knockout, but changes to the beta and gamma clusters were not observed.

## Discussion

Gene silencing at the Xi is remarkably stable, involving multiple redundant gene silencing mechanisms that ensure silencing is maintained throughout all subsequent somatic cell divisions. Stability of the imprinted Xi in extraembryonic material is compromised upon loss of the Polycomb group protein Embryonic Ectoderm Development (EED) [72,73], but there are few other examples of Xi instability due to a single gene loss. In fact, global reactivation of the Xi has only been achieved through fusion of female mammalian cells with mouse embryonal carcinoma cells [74-77] or via reprogramming mouse female cells to pluripotency through forced expression of transcription factors [78]. Therefore, if WSTF is involved in maintaining chromatin states at the Xi, it is not surprising that the loss of WSTF did not result in an obvious wave of X-linked gene reactivation and loss of bonafide Xi markers, such as XIST or H3K27me3. Genes that are reactivated from the Xi are unlikely to be expressed at levels dramatically different from their Xa counterpart. Consequently, the anticipated change in gene expression would likely be less than two fold. However, reassessing the data for 1.5 fold change did not show a bias toward increase in X-linked gene expression levels compared to the autosomes (data not shown). Alternatively, however, these observations can be interpreted to suggest that perhaps the transient association of WSTF with the chromosome, as the Xi DNA is replicated [15], is unrelated to maintenance of XCI.

WSTF, as part of the WICH complex, has been shown to transiently associate with the Xi prior to γ-H2A.X and BRCA1 [15]. The fact that BRCA1 and γ-H2A.X continue to transiently associate with the Xi when WSTF is absent suggests that the two cell-cycle linked events are not coupled. Indeed, we have previously speculated that WICH may transiently associate with the newly replicated DNA in order to reestablish the basal state of H2A.X-Y142ph. This theory supports the idea that WSTF recruitment to the Xi is independent of γ-H2A.X and BRCA1 association since the ground state of H2A.X-Y142ph is set independently of DNA damage [21].

Intriguingly, almost one quarter of *BAZ1B* knockout cells displayed numerous, large, DAPI-dense blocks. Consistent with the DAPI staining, the blocks are characterized by constitutive heterochromatin-associated proteins, including HP1, HMG-I/Y, and MBD1, and by the covalent histone modification H3K9me3. In addition, the HP1-interacting protein SMCHD1 [58], was also seen localizing to the blocks. While the blocks did not overlap with nucleoli, they were often seen abutting these nuclear structures, as indicated by NOR DNA FISH and MYBBP1A IF; markers of nucleoli [15]. Prior studies have shown that, in addition to rDNA-containing regions of the acrocentric chromosomes, heterochromatin domains of most human chromosomes (including regions found at telomeres, centromeres, and chromosome arms), associated with nucleoli [79]. Perhaps, perinucleolar regions are sites of heterochromatin maintenance. Indeed, in mouse, the binding partner of Wstf, Snf2h, transiently accumulates in a perinucleolar region as the mouse Xi is replicated [80]. Perhaps, in *BAZ1B* knockout cells, checks on heterochromatin formation are compromised resulting in the inappropriate spread into genomic regions not normally associated with heterochromatin. The obvious gain in the large blocks of heterochromatin support a model in which WSTF assists in maintaining chromatin states, and in its absence, too much heterochromatin forms [17].

The size and number of DAPI-dense blocks in *BAZ1B* knockout cells indicates that the gain of heterochromatin in affected cells is substantial. Previously, it was shown that artificially reducing WSTF protein levels by RNA interference, was associated with lower levels of transcription, as measured by monitoring the levels of bromouridine incorporation into RNA [18]. By microarray analysis, a large number of genes consistently showed a reduction in expression (342) in three independent mutants. This change could be attributed to the gain in heterochromatin, although secondary effects, such as silencing of a key transcription factor, cannot be ruled out. Of interest, the reduced expression was not global, impacting only a subset of all genes. This suggests that if WSTF is acting to reestablish appropriate chromatin states post DNA replication, as postulated [17], it is either only acting at specific regions of the genome, as most genes are unaffected by the proteins loss, or that certain regions of the genome are more vulnerable to inappropriate heterochromatin spread/assembly than others.

We also observed a large number of genes that showed an increase in expression (173) in all three *BAZ1B* knockout clones. This is consistent with WSTF’s previously established role as a regulator of transcription, capable of activating or silencing genes [62]. Notably, loss of a single *BAZ1B* allele was sufficient to impact a subset of those genes affected in the knockouts, indicating that some genes are more sensitive to WSTF levels than others. This has important implications for *BAZ1B* hemizygosity in WBS, and our heterozygous targeted clones provide an important model in which to pursue the contribution of WSTF to the phenotype in this complex contiguous gene deletion disorder.

Some genes showed reactivation from a silent state in the mutants. In contrast, relatively few genes showed the opposite trend of complete silencing in the mutants. It is possible that within the pool of cells assessed, complete silencing might only be observed in cells with DAPI-dense blocks that represent fewer than 25% of the total cell population. In these cells, genes embedded in the blocks or within their vicinity might be fully silenced; yet not in the 75% of cells that appear normal. Continuing with this notion, it is possible that the actual number of genes impacted by WSTF loss might be greater than our microarray data would otherwise suggest. If some genes show increases or decreases in gene expression that are close to, but greater than, our 2-fold cutoff *only* in DAPI-dense block nuclei, these changes might be reduced to below the cutoff by a lack of change in the majority non-DAPI-dense block cells.

Despite the extensive size of the DAPI blocks, we did not see an increase in nuclear levels of H3K9me3. Therefore, an alternative explanation for the reactivation of genes could be an indirect effect, as H3K9me3 is redistributed to the blocks, essentially reducing local repressive effects elsewhere.

The dynamic nature of the blocks is intriguing. Cells that acquire blocks do not undergo apoptosis and the cell cycle is apparently unaffected. Single cell cloning indicates that block formation is reversible, and that the overall number of cells with blocks remains relatively constant within an actively dividing population and between independent mutants. Live cell imaging confirms that block appearance is not linked to the cell cycle, and indicates that blocks are essentially immobile, and do not prevent cells from proceeding through mitosis. Reintroduction of wild-type WSTF resolves the blocks, and therefore provides a powerful manipulable system in which to investigate regions of the WSTF protein that are required for this activity.

## Conclusions

In this study, we have explored the impact of WSTF loss. Our findings emphasize that many aspects of the function of WSTF remain enigmatic. Identifying the common characteristics of the DNA sequences in the DAPI-dense blocks may provide clues as to how WSTF contributes to gene regulation. It is important for future studies to examine the mechanism of WSTF action so that we may fully understand the role that WSTF plays in nuclear processes and its contribution to WBS. In addition, our heterozygous mutant cells provide a useful model to investigate the effects of *BAZ1B* hemizgosity and WSTF haploinsufficiency in the absence of the loss of the other 27 genes seen in WBS.

## Methods

### Cell culture

The cell line used to generate the knockout clones was hTERT-RPE1, a 46, XX telomerase-immortalized human retinal pigment epithelial cell line (Clontech, Laboratories, Inc., Mountain View, CA, USA: No. C4000-1). The cells were maintained according to the supplier’s recommendations.

### Generating *BAZ1B* knockout cell lines

The targeting template for homology mediated repair was generated as follows. The left homology arm (HA) was amplified by PCR with the primers LHA-F2 and LHA-R2 (Additional file 7) using RPE1 genomic DNA as a template. The same was done for the right HA with the primers RHA-F1 and RHA-R1 (Additional file 7). The PCR products for the left and right HAs were TA cloned into pDRIVE (Qiagen, Valencia, CA, USA). Plasmid DNA was harvested using the Nucleospin Plasmid kit (Machery-Nagel, Bethlehem, PA, USA), before sequence verifying. The insert of the left HA was excised from the pDRIVE vector using *Not*I and *Xba*I, and the right HA was excised using *Xho*I and *Mlu*I. The pSEPT cassette was digested with *Xba*I and *Xho*I and the pBC-KO-tk-A vector (Chadwick, manuscript in preparation) was digested with *Not*I and *Mlu*I. All digests were separated on agarose gels (Agarose Unlimited, FL, USA), and inserts excised and purified using a Nucleospin Gel and PCR Clean-up kit (Machery-Nagel, Bethlehem, PA, USA). The left HA, pSEPT cassette, and right HA, were ligated overnight at 16°C into pBC-KO-tk-A vector using T4 DNA ligase (NEB, Ipswich, MA, USA). The ligation was transformed into NEB-5alpha chemically competent E. coli cells according to the supplier’s recommendations (NEB, Ipswich, MA, USA). Plasmid DNA was prepared using the Plasmid Plus Midi kit (Qiagen, Valencia, CA, USA). The plasmid DNA was linearized by digestion with *Asc*I restriction enzyme, and gel purified. To generate *BAZ1B* knockout cell lines, we introduced ZFN expression constructs (Sigma-Aldrich, St. Louis, MO, USA: Cat No. CSTZFN-1KT) and the linearized repair template by Nucleofection (Lonza Group Ltd, Basel, Switzerland). Twenty-four hours post-Nucleofection, cells were seeded into four 96-well plates in media supplemented with neomycin (150 μg/mL). After approximately two weeks, clones were expanded and genomic DNA isolated using a Wizard SV 96 Genomic DNA Purification System kit (Promega, Madison, WI, USA). Correctly targeted clones were screened on the left and right sides by PCR between a primer to the genomic locus, but distal/proximal to the HA, and a primer located in the pSEPT cassette. The screening pairs were Left-Screen-F2 with pSEPT-R1 and pSEPT F2 with Right-Screen-R1 (Additional file 7). Clones that generated the correct sized PCR product using these two screening pairs were tested for NHEJ by PCR using the primers *BAZ1B* Sigma-F and *BAZ1B* Sigma-R (Additional file 7). If a product was generated, it was sequenced to determine if NHEJ had resulted in insertions or deletions. All restriction enzymes were obtained from NEB (Ipswich, MA, USA).

### WSTF rescue

A full-length wild-type human myc-tagged WSTF expression construct was obtained from Origene (Rockville, MD, USA: Cat.No. RC216159). The expression construct was introduced into *BAZ1B* knockout cells by Nucleofection using a 4D-Nucleofector (Lonza Group Ltd, Basel, Switzerland) and solution SF on program EN-138 according to the manufacturers recommendations.

### Antibodies

#### ***Immunofluorescence***

Rabbit anti-WSTF antibodies were obtained from Cell Signaling Technology (Danvers, MA, USA: Cat No. 2152) and Bethyl Laboratories (Montgomery, TX, USA: Cat No. A300-446A). Rabbit anti-SMCHD1 (Cat No. A302-872A) was also purchased from Bethyl Laboratories (Montgomery, TX, USA). Rabbit anti-CDT1 (Cat No. 10/2012), rabbit anti-SETDB1 (Cat No. 06/2012), rabbit anti-CENP-A (Cat No. 2186S), mouse anti-TIF1B (Cat No. 5868S), and mouse monoclonal antibodies to myc (Cat No. 2276S) were purchased from Cell Signaling Technologies (Danvers, MA, USA). Mouse monoclonal antibodies BRCA1 (Cat No. sc-6954) were obtained from Santa Cruz Biotech (Dallas, Texas, USA) as were goat anti-SAP18 (Cat No. sc-8473), mouse anti-Histone H1(sc-8030), goat anti-MBD1 (Cat No. sc-9395), goat anti-MBD2 (Cat No. sc-12444), goat anti-MBD3 (Cat No. sc-9402), goat anti-RbAp46 (Cat No. sc-8272), goat anti-RbAp48 (Cat No. sc-8270), goat anti-ATRX (Cat No. sc-10080), rabbit anti-SIN3A (Cat No. sc-994), rabbit anti-SIN3B (Cat No. sc-768), rabbit anti-Mi2 (Cat No. sc-1564), and rabbit anti-HMG-I/Y (Cat No. sc-1564). Antibodies to Mouse monoclonal antibodies to phospho-H2A.X (Serine-139) (Cat No. 05–636) and SNF2H (Cat No. 05–698) were obtained from Millipore (Billerica, MA, USA), as were rabbit polyclonal antibodies to EZH2 (Cat No. 07–400), histone H3 trimethylated at lysine-27 (Cat No. 07–449), histone H3 phosphorylated at serine 10 (Cat No. 06–570), histone H3 dimethylated at lysine 4 (Cat No. 07–0030), histone H3 acetylated at lysine 9 (Cat No. 06–942), and histone H3 trimethylated at lysine 9 (Cat No. 07–523). Mouse anti-HP1α (Cat No. MAB3584), anti-HP1β (Cat No. MAB3448), and anti-HP1γ (Cat No. MAB3450) were obtained from Chemicon International (EMD Millipore, Billerica, MA, USA). Mouse anti-PML (Cat No. ab96051) antibody was purchased from Abcam (Cambridge, MA, USA). Mouse anti-MYBBP1A (Cat No. SAB1400390) was obtained from SIGMA-Aldrich (St. Louis, MO, USA). Rabbit anti-macroH2A.1 antibody was described previously [81]. Alexa-Fluor^®^ conjugated secondary antibodies were obtained from Life Technologies Corporation (Invitrogen, Grand Island, NY, USA).

### DNA and RNA FISH probes

Direct-labeled FISH probes were prepared using a Nick Translation kit with SpectrumGreen dUTP according to the manufacturer’s recommendations (Abbott Molecular, Abbott Park, IL, USA). P1-artificial chromosome (PAC) RP5-1174A5, which contains rDNA and detects the Nucleolar Organizing Regions (NOR), was obtained from Dr. P. Finelli (Finelli et al. 2012). The pan-centromeric probe (Cat No. 1695-F-02) was purchased from Cambio (Cambridge, UK). The XIST RNA-FISH probe was as described previously [15].

### Western blotting

The protein samples for Western blotting were prepared by lysing approximately one million cells in RIPA buffer according to standard procedures [82]. Samples were separated on 4-20% acrylamide gels at 200 V. Histone samples for Western blotting were prepared according to Abcam’s histone extraction protocol (http://www.abcam.com/index.html?pageconfig=resource&rid=11410). Samples were separated on AnykD acrylamide gels (Biorad, Hercules, CA, USA) at 200 V. All SDS-PAGE gels were transferred to pre-equilibrated 0.2 μ PVDF (Biorad, Hercules, CA, USA) using a BioRad Turbo Transfer apparatus. The PVDF membranes were blocked with 5% non-fat dry milk in 0.1% PBS-Tween 20 for 30 minutes and washed twice in 0.1% PBS-Tween 20/1% igepal for 15 minutes each. Primary antibodies were diluted in blocking solution and either incubated with gentle rocking overnight at 4C (WSTF Cell Signaling Technologies and Bethyl Laboratories antibodies) or for 1 hour at room temperature (all other antibodies). The membranes were washed twice as described above. The membranes were incubated with horseradish peroxidase-conjugated (HRP) secondary antibodies diluted in blocking solution for 1 hour at room temperature with gentle rocking. The membranes were washed as previously described. Detection of HRP was achieved by incubating membranes with equal volumes of ECL Solution A and Solution B (GE Healthcare, Pittsburgh, PA, USA) for 3 minutes at room temperature. The two WSTF antibodies described above were used. Rabbit anti-B-Actin (Cat No. 11/2012) was purchased from Cell Signaling Technology (Danvers, MA, USA). The rabbit anti-H3K9me3 antibody described above for IF analysis was used for Western analysis. Rabbit anti-Histone 4 (Cat No. 07–108) was obtained from Millipore (Billerica, MA, USA). HRP anti-rabbit secondary antibody was obtained from Cell Signaling Technology (Danvers, MA, USA: Cat No. 7074S).

### DNA and RNA FISH and indirect immunofluorescence

To prepare slides for IF and FISH studies, approximately 2×10^5^ cells were grown directly on standard microscope slides. IF was performed as described [15]. DNA was counterstained using ProLong^®^ Gold antifade reagent supplemented with 4′,6-diamidino-2-phenylindole (DAPI) (Invitrogen, Grand Island, NY, USA). For IF combined with DNA FISH, IF was first performed. The FISH probes were denatured in the presence of human Cot-1 DNA (Invitrogen, Grand Island, NY, USA) at 78°C for 10 minutes followed by 30 minutes at 37°C to block repetitive sequences before applying to the slides. Slides were then treated as previously described [15]. Antifade containing DAPI was added to the slides, followed by a cover slip. IF combined with RNA FISH was performed essentially as described [83].

### Image acquisition

Images were collected using a DeltaVision pDV (GE Healthcare, Pittsburgh, PA, USA). Delta Vision images were deconvolved with softWoRx 3.7.0 (Applied Precision, Issaquah, WA, USA) and compiled with Adobe Photoshop CS2 (Adobe Systems, San Jose, CA, USA). Live cell imaging videos were recorded using an Andor Revolution Spinning Disk Laser Confocal microscope and an Andor 897 EMCCD camera (Andor Technology plc. Belfast, UK). Live cell imaging data was processed using Andor iQ2 software.

### HP1-GFP Expression constructs

GFP-tagged HP1-α, β and γ expression constructs were generated by excision with *Eco*RI of the full ORF inserts from previously described pcDNA3.1-CT-Myc constructs [84], into the *Eco*RI site of pcDNA3.1-CT-GFP (Invitrogen, Grand Island, NY, USA).

### Fluorescence activated cell sorting (FACS) analysis

For FACS analysis, an asynchronously growing population of 1 × 10^6^ cells were stained with propidium iodide [85] and flow sorted using a FACSCanto (BD Bioscience, San Jose, CA, USA). Cell cycle profiles were generated using FACSCanto software.

### Microarray gene expression analysis

Microarray expression analysis was performed on parental cells as well as three independent knockout mutants and one heterozygously targeted clone. For each sample, total RNA was extracted from three independent cell pellets isolated at different time points. Total RNA was extracted from cells using the RNeasy Mini Kit from Qiagen (Valencia, CA, USA). Eluted RNA was treated with DNaseI (NEB, Ipswich, MA, USA) before cleaning and purifying the RNA using an RNeasy Mini Kit column (Qiagen, Valencia, CA, USA) according to the manufacturers recommendations. RNA quality was assessed by agarose gel electrophoresis and absence of contaminating DNA was confirmed by PCR with primers to the large tandem repeat DXZ4 [86]. Single stranded cDNA was prepared using the High Capacity cDNA Reverse Transcription Kit (Invitrogen, Grand Island, NY, USA: Cat. No. 4368814). The cDNA was then provided to the FSU-Nimblegen Microarray Facility for labeling, hybridization, and washing. Probes were hybridized onto a Human Expression 12×135K array (Roche-Nimblegen, Indianapolis, IN, USA: 05543789001). Hybridization data was analyzed using DNA ArrayStar version 4 (DNASTAR, Madison, WI, USA). The three replicates of RPE1 were compared to one another. Transcript targets were included if their confidence level was ≥ 95% based on a p-value of p < 0.05. This was done for each of the mutant clones. Replicate sets of each of the mutants were compared to the RPE1 replicate set. In order to be included, greater than two fold change in expression was required, with a confidence level of ≥95% based on a p-value of p < 0.05. Additionally, transcript targets that did not meet these criteria in all three of the independent mutants were excluded. Microarray expression data has been submitted to the Gene Expression Omnibus, and has been assigned accession number GSE47390.

### Quantitative RT-PCR Analysis

All qRT-PCR was performed on a CFX96 (Biorad, Hercules, CA, USA) using EvaGreen 2X qPCR Mastermix (ABM, Richmond, BC, Canada: Cat No. Mastermix-S). The two-step amplification cycle began with a 10 minute incubation at 95C, followed by 40 cycles of 15 seconds at 95C and 30 seconds at 60C. Fluorescence was measured at the end of each of the 40 cycles. Afterwards, a melt curve was generated by measuring the fluorescence after heating for 5 seconds in 0.5C increments, beginning at 65C and ending at 95C. qRT-PCR analysis of *BAZ1B* used Quantitect primer assay QT00072044 along with *GAPDH* assay QT01192646. Both were obtained from Qiagen (Valencia, CA, USA).

### Standard RT-PCR Analysis

Standard RT-PCR was performed using OneTaq (NEB, Ipswich, MA, USA). The cDNA samples were denatured at 94°C for 30 seconds, amplified through 40 cycles of 30 seconds at 94°C, 30 seconds at 58°C, and 30 seconds at 68°C, and finally held at 15°C. Details on the primers used can be found in Additional file 7.

## Abbreviations

BAZ1B: Bromodomain adjacent to zinc finger domain 1B; BRCA1: Breast cancer 1 gene; DAPI: 4′,6-diamidino-2-phenylindole; FACS: Fluorescence activated cell sorting; FISH: Fluorescence *in situ* hybridization; GFP: Green fluorescent protein; γ-H2A.X: Histone H2A.X phosphorylated at serine 139; H2A.X: Histone H2A variant X; H2A.X-Y142ph: H2A.X phosphorylated at tyrosine 142; H3K4me2: Histone H3 dimethylated at lysine 4; H3K9Ac: Histone H3 acetylated at lysine 9; H3K9me3: Histone H3 trimethylated at lysine 9; H3K27me3: Histone H3 trimethylated at lysine 27; H3S10ph: Histone H3 phosphorylated at serine 10; HA: Homology arm; HP1: Heterochromatin protein 1; IF: Immunofluorescence; mH2A1: macroH2A1; NHEJ: Non homologous end joining; NOR: Nucleolar organizing region; ORF: Open reading frame; PCNA: Proliferating cell nuclear antigen; PCR: Polymerase chain reaction; PML: Promyelocytic leukemia; qRT-PCR: Quantitative real time polymerase chain reaction; rDNA: Ribosomal deoxyribonucleic acid; RNAi: RNA interference; RPE1: hTERT-Retinal pigment epithelial; RT-PCR: Real time polymerase chain reaction; SEPT: Synthetic exon promoter trap; SMCHD1: Structural maintenance of chromosomes flexible hinge domain containing 1; SNF2H: Sucrose nonfermenting 2 homolog; Try142: Tyrosine 142; WBS: Williams-Beuren syndrome; WICH: WSTF-ISWI chromatin remodeling complex; WSTF: Williams syndrome transcription factor; XCI: X chromosome inactivation; Xi: Inactive X chromosome; XIST: X-inactive specific transcript; ZFN: Zinc finger nuclease.

## Competing interests

Both authors declare they have no competing interests.

## Authors’ contributions

Conceived and designed the experiments: AECC, BPC. Performed the experiments: AECC, BPC. Analyzed the data: AECC, BPC. Contributed reagents/materials/analysis tools: AECC, BPC. Wrote the paper: AECC, BPC. Both authors read and approved the final manuscript.

## Supplementary Material

Additional file 1 **Representative IF images of chromatin proteins relative to the DAPI-dense blocks and the normal distribution of H3K9me3, HP1 and SMCHD1 in parental RPE1 cells.** Top panels show representative IF images of *BAZ1B* -/- cells with DAPI-dense blocks, showing the distribution of various chromatin proteins (red or green). Bottom panels show the normal distribution of H3K9me3, HP1 and SMCHD1 in parental RPE1 nuclei. The nucleus is counterstained with DAPI (blue). Click here for file

Additional file 2 **Movie clip of a HP1β-GFP Nucleofected *BAZ1B *-/- heterochromatin block containing cell entering mitosis uninhibited.** Movie shows a cluster of three *BAZ1B* -/- clone D5 nuclei that are positive for HP1β-GFP blocks. Toward the end of the movie, the middle cell enters and exits mitosis. The movie represents a time lapse of 21 hours and 50 minutes with images captured every 5 minutes under 40X magnification. Note: The duration of the cell cycle is potentially slowed due to frequent exposure to the illumination source. Click here for file

Additional file 3 **Movie clip of a HP1β-GFP Nucleofected *BAZ1B* -/- heterochromatin block containing cell entering mitosis and giving rise to a block-containing daughter cell.** Movie shows a *BAZ1B* -/- clone D5 nuclei that is positive for HP1β-GFP blocks (top left), that passes through mitosis and gives rise to a block-containing nucleus. The blocks appear to resolve toward the end of the movie leaving the Xi-associated HP1β-GFP as the only remaining obvious block. For comparison, an HP1β-GFP positive non-block containing nucleus shows only the Xi-associated HP1β-GFP (lower central portion of movie). The movie represents a time lapse of 62 hours and 20 minutes with images captured every 10 minutes under 60X magnification. Note: The duration of the cell cycle is potentially slowed due to frequent exposure to the illumination source. Click here for file

Additional file 4 **Movie clip of a HP1β-GFP Nucleofected *BAZ1B* -/- heterochromatin block containing cell showing the static nature of the block positions in the nucleus.** Movie shows a *BAZ1B* -/- clone D5 nuclei that is positive for HP1β-GFP blocks, that develops intense blocks that show no obvious movement despite the mobility of the cell. The movie represents a time lapse of 62 hours and 20 minutes with images captured every 10 minutes under 60X magnification. Note: The duration of the cell cycle is potentially slowed due to frequent exposure to the illumination source. Click here for file

Additional file 5 **Excel file listing genes that consistently change expression by >2-fold in *BAZ1B* -/- cells.** File contains tabs for all data combined, as well as gene expression for each chromosome individually. Click here for file

Additional file 6 **Excel file listing genes that consistently change expression by >2-fold in *BAZ1B* +/- cells that are shared with *BAZ1B* -/- cells.** Click here for file

Additional file 7 Table listing all oligonucleotides used in this study. Click here for file
