# Supplementary figures and images for: Loss of WSTF results in spontaneous fluctuations of heterochromatin formation and resolution, combined with substantial changes to gene expression
Source: BMC Genomics. 2013 Oct 29;14:740. doi: 10.1186/1471-2164-14-740 (PMC3870985; doi:10.1186/1471-2164-14-740)

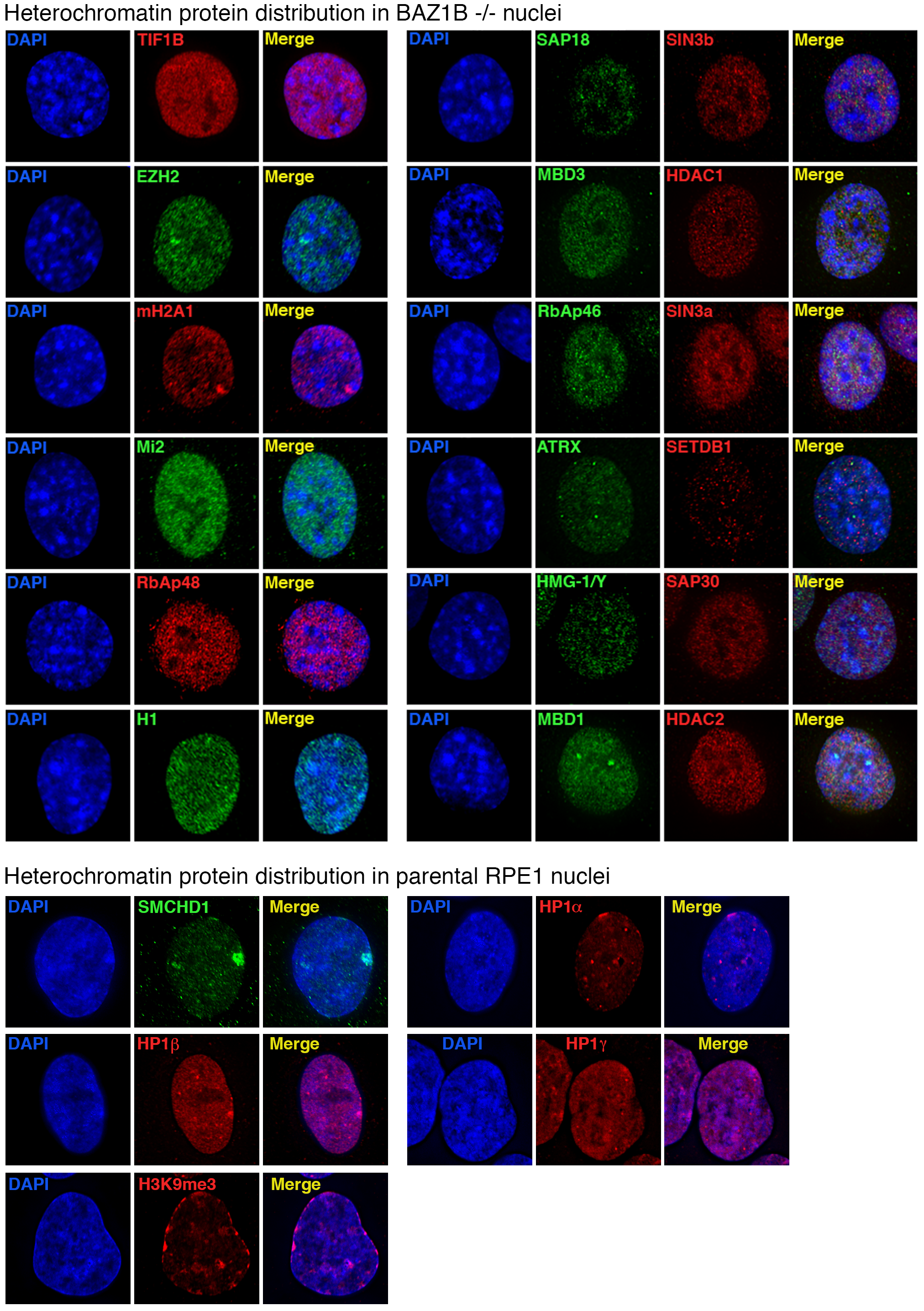

Supplement: Additional file 1 — Representative IF images of chromatin proteins relative to the DAPI-dense blocks and the normal distribution of H3K9me3, HP1 and SMCHD1 in parental RPE1 cells. Top panels show representative IF images of BAZ1B -/- cells with DAPI-dense blocks, showing the distribution of various chromatin proteins (red or green). Bottom panels show the normal distribution of H3K9me3, HP1 and SMCHD1 in parental RPE1 nuclei. The nucleus is counterstained with DAPI (blue). [file 1471-2164-14-740-S1.tiff]
